# HER2DX *ERBB2* mRNA score in first-line advanced HER2-positive breast cancer treated with chemotherapy, trastuzumab, and pertuzumab

**DOI:** 10.1038/s41523-025-00753-8

**Published:** 2025-04-25

**Authors:** Rodrigo Sánchez-Bayona, Olga Martínez-Sáez, Denys Romero-Romero, Elia Seguí, Esther Carcelero, Pablo Tolosa, Jesús Soberino, Manuel Alva, Tomás Pascual, Laura Lema, Isabel Garcia-Fructuoso, Maria Angeles Cobos-Fernandez, Maria Rey, Luis Manso, Angela Aguirre, Ainhoa Madariaga, Valeria Sirenko, Cristina González-Deza, Paula Blasco, Astrid Mayhua, Oleguer Castillo, Patricia Galván, Esther Sanfeliu, Guillermo Villacampa, Wesley Buckingham, Mercedes Marín-Aguilera, Laia Paré, Patricia Villagrasa, Charles M. Perou, Julia Maues, Fara Brasó-Maristany, Eva Ciruelos, Aleix Prat

**Affiliations:** 1 Hospital Universitario 12 de Octubre, Madrid, Spain; 2 SOLTI Breast Cancer Research Group, Barcelona, Spain; 3 Translational Genomics and Targeted Therapies in Solid Tumors, August Pi I Sunyer Biomedical Research Institute (IDIBAPS), Barcelona, Spain; 4 Medicine Department, University of Barcelona, Barcelona, Spain; 5 Cancer Institute and Blood Disorders, Hospital Clinic, Barcelona, Spain; 6 Pharmacy Department, Hospital Clinic, Barcelona, Spain; 7 Breast Cancer Unit, IOB-QuirónSalud, Barcelona, Spain; 8 Pathology Department, Hospital Clínic de Barcelona, Barcelona, Spain; 9 Statistics Unit, Vall d’Hebron Institute of Oncology (VHIO), Barcelona, Spain; 10 Reveal Genomics, Barcelona, Spain; 11 GRASP, Baltimore, MD USA; 12 Centro Integral Oncológico Clara Campal HM (CIOCC), Madrid, Spain

**Keywords:** Breast cancer, Predictive markers

## Abstract

In advanced HER2-positive breast cancer, the standard taxane-trastuzumab-pertuzumab (THP) regimen faces competition from new therapies, emphasizing the need for biomarkers to guide treatment. This study evaluates the HER2DX *ERBB2* mRNA score as a prognostic predictor, aiming to tailor treatment strategies. We retrospectively analyzed 94 patients treated with the THP regimen between 2010 and 2024. The HER2DX *ERBB2* mRNA score was categorized as low (*n* = 14), medium (*n* = 20), or high (*n* = 60), and its correlation with progression-free survival (PFS) and overall survival (OS) was assessed using Cox regression models. The median follow-up was 31.5 months. Patients with *ERBB2*-high scores had significantly better median PFS (33.9 vs. 10.6 months, hazard ratio [HR] = 0.40, 95% CI: 0.24–0.69, *p* < 0.001) and OS (not reached vs. 30.8 months, HR = 0.26, 95% CI: 0.13–0.49, *p* < 0.001) compared to *ERBB2*-low patients. Based on these findings, further validation of this biomarker in tumor samples from the CLEOPATRA phase III trial is ongoing, which could help optimize treatment strategies in this population.

## Introduction

In the first-line treatment of advanced HER2-positive (HER2+) breast cancer, the standard regimen of taxane-trastuzumab-pertuzumab (THP) provides a median progression-free survival (PFS) of approximately 18–21 months and a median overall survival (OS) of around 57–65 months^1,2^. While THP remains a cornerstone therapy, antibody–drug conjugates (ADCs) are increasingly challenging this standard. ADCs, such as trastuzumab deruxtecan (T-DXd), have demonstrated superior outcomes compared to standard treatments in patients previously treated with chemotherapy and anti-HER2 therapy^3–5^. The ongoing DestinyBreast-09 trial (NCT04784715)^6^, exploring THP versus T-DXd with or without pertuzumab in the first-line setting, reflects this shift. These developments underscore the critical need for robust biomarkers to guide treatment decisions in advanced HER2+ breast cancer.

Efforts to identify prognostic factors in patients treated with THP have yielded only a few clinical and analytical markers—such as non-visceral disease, good performance status, oligometastatic disease, and low baseline neutrophil/lymphocyte ratio—linked with longer PFS^7,8^. However, the correlation between these factors and OS remains unclear, necessitating novel approaches in the context of precision oncology.

Transcriptomics in HER2+ breast cancer has significantly advanced the understanding of breast cancer’s complexity and heterogeneity^9–11^, enabling more precise diagnostic tools. To date, no biomarker has been validated for predicting prognosis or treatment efficacy in advanced HER2+ breast cancer. However, in early-stage HER2+ breast cancer, the 27-gene HER2DX genomic assay, which provides a pathological complete response (pCR) likelihood score and a risk score, has demonstrated both prognostic and predictive value^12–19^. In addition, HER2DX provides the *ERBB2* mRNA score, offering a continuous assessment of *ERBB2* mRNA levels associated with HER2 protein expression, reported on a scale from 1 to 99^15^. It categorizes patients into three groups: *ERBB2*-low (1–32), *ERBB2*-medium (33–50), and *ERBB2*-high (51–99). These cutoffs were trained to predict clinical HER2 status based on the American Society of Clinical Oncology/College of American Pathologists (ASCO/CAP) guidelines^20^. The 33 cutoff in the score distinguishes between *ERBB2*-low and *ERBB2*-medium/high, correlating with clinical HER2-negative versus HER2+ status, while the 51 cutoff separates *ERBB2*-medium from *ERBB2*-high, corresponding to the lower 33% of HER2DX *ERBB2* expression levels in HER2+ breast cancer^15^.

Given the potential of the HER2DX *ERBB2* mRNA score as a predictive biomarker, particularly in the context of THP treatment, our study aims to validate the ability of the HER2DX *ERBB2* mRNA score to predict survival outcomes in patients with advanced HER2+ breast cancer treated with first-line THP. By identifying patients who may benefit more from THP versus emerging therapies like T-DXd, we seek to refine therapeutic approaches in this evolving landscape.

## Results

### Clinicopathological features of the patients

**Fig. 1:**
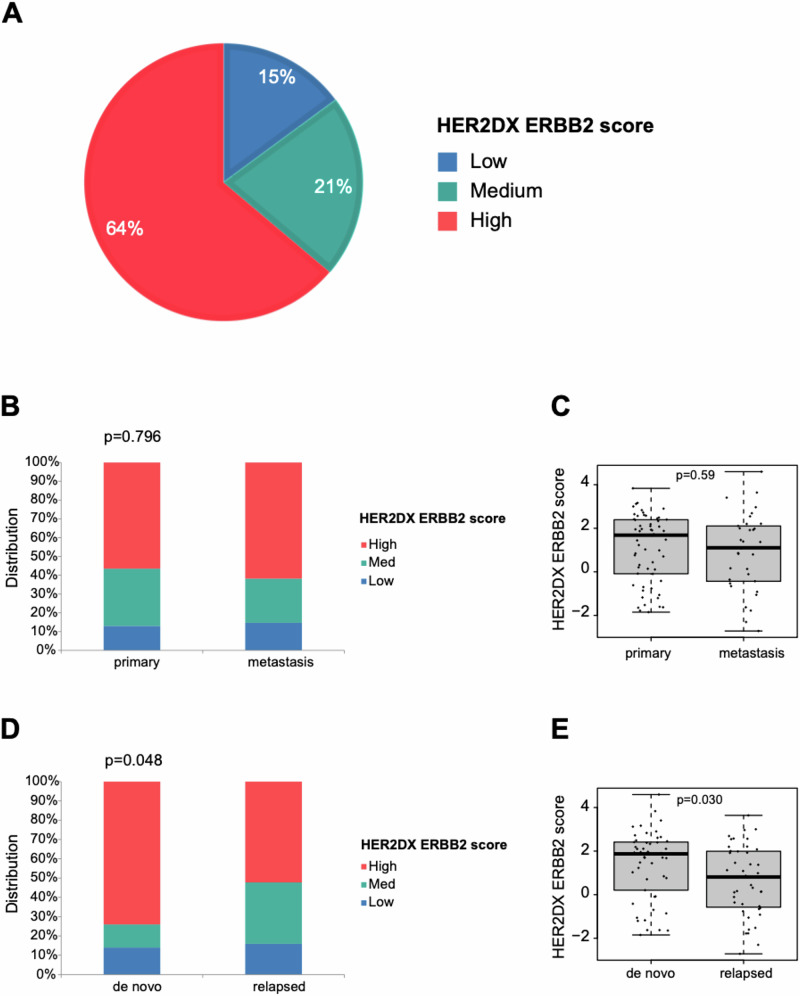
Distribution of HER2DX *ERBB2* mRNA score. **A** Distribution of HER2DX *ERBB2* mRNA score groups across the 94 patients. **B** Distribution of HER2DX *ERBB2* mRNA score groups in primary and metastatic tumor. *P*-value was determined using Fisher’s exact test. **C** HER2DX *ERBB2* mRNA score in primary and metastatic tumors. *P*-value was determined using an unpaired *t*-test. **D** Distribution of HER2DX *ERBB2* mRNA score groups in de novo and relapsed disease. The *P*-value was determined using Fisher’s exact test. **E** HER2DX *ERBB2* mRNA score in de novo and relapsed disease. The *P*-value was determined using an unpaired *t*-test.

A total of 94 patients diagnosed with HER2+ metastatic breast cancer and treated with THP were included in this study. The baseline characteristics of the cohort are summarized in Table 1. The median follow-up was 31.5 months (1.5–162.5 months). Our cohort consisted of hormone receptor-positive cases (61.7%), patients with visceral disease (73.4%), brain metastasis (13.8%), and de novo metastatic disease (53.2%). Most patients were treated with paclitaxel (*n* = 79, 84%), and the remaining with docetaxel (*n* = 15, 16%). Based on pre-specified cutoffs for the HER2DX *ERBB2* mRNA score, the cohort distribution was 14.9% in the low group, 21.3% in the medium group, and 63.8% in the high group (Fig. 1A). No significant differences in HER2DX *ERBB2* mRNA score were observed between primary (*n* = 60) and metastatic tumor (*n* = 34) tissues as a categorical or continuous variable (Fig. 1B, C). However, the HER2DX *ERBB2* mRNA score was statistically significantly higher in patients with de novo metastatic disease than those with recurrent disease (Fig. 1D, E). The HER2DX *ERBB2* score, analyzed as a continuous variable, was significantly associated with HER2 IHC (Supplementary Fig. 1a). Among HER2 IHC 3+ tumors (*n* = 63), the distribution of HER2DX *ERBB2* scores as a categorical variable showed that 76.2% were classified as high, 17.5% as medium, and 6.3% as low. In HER2 IHC 2+ tumors (*n* = 24), the distribution was 50.0% high, 20.8% medium, and 29.2% low. Notably, in HER2 IHC 0 (*n* = 3) and 1+ (*n* = 2) tumors, no cases were classified as high (Supplementary Fig. 1b).

**Table 1:** Baseline characteristics of the patient cohort (*n* = 94)

	*N*	%
Source		
Hospital Univ. 12 Octubre	35	37.2
Hospital Clinic Barcelona	59	62.8
Type of biopsy analyzed		
Primary	60	63.8
Metastatic	34	36.2
HER2 IHC status		
3+	63	67.0
other	31	33.0
Hormone receptor status		
Negative	36	38.3
Positive	58	61.7
Type of advanced disease		
De novo	50	53.2
Relapse	44	46.8
Menopausal status		
Premenopausal	20	21.3
Postmenopausal	72	76.6
Gender		
Female	92	97.9
Male	2	2.1
Metastatic pattern		
Visceral disease	69	73.4
Brain disease	13	13.8
Bone-only disease	14	14.9
Metastatic burden		
<3 metastatic sites	59	62.8
≥3 metastatic sites	35	37.2

### HER2DX *ERBB2* mRNA score and clinical endpoints

**Fig. 2:**
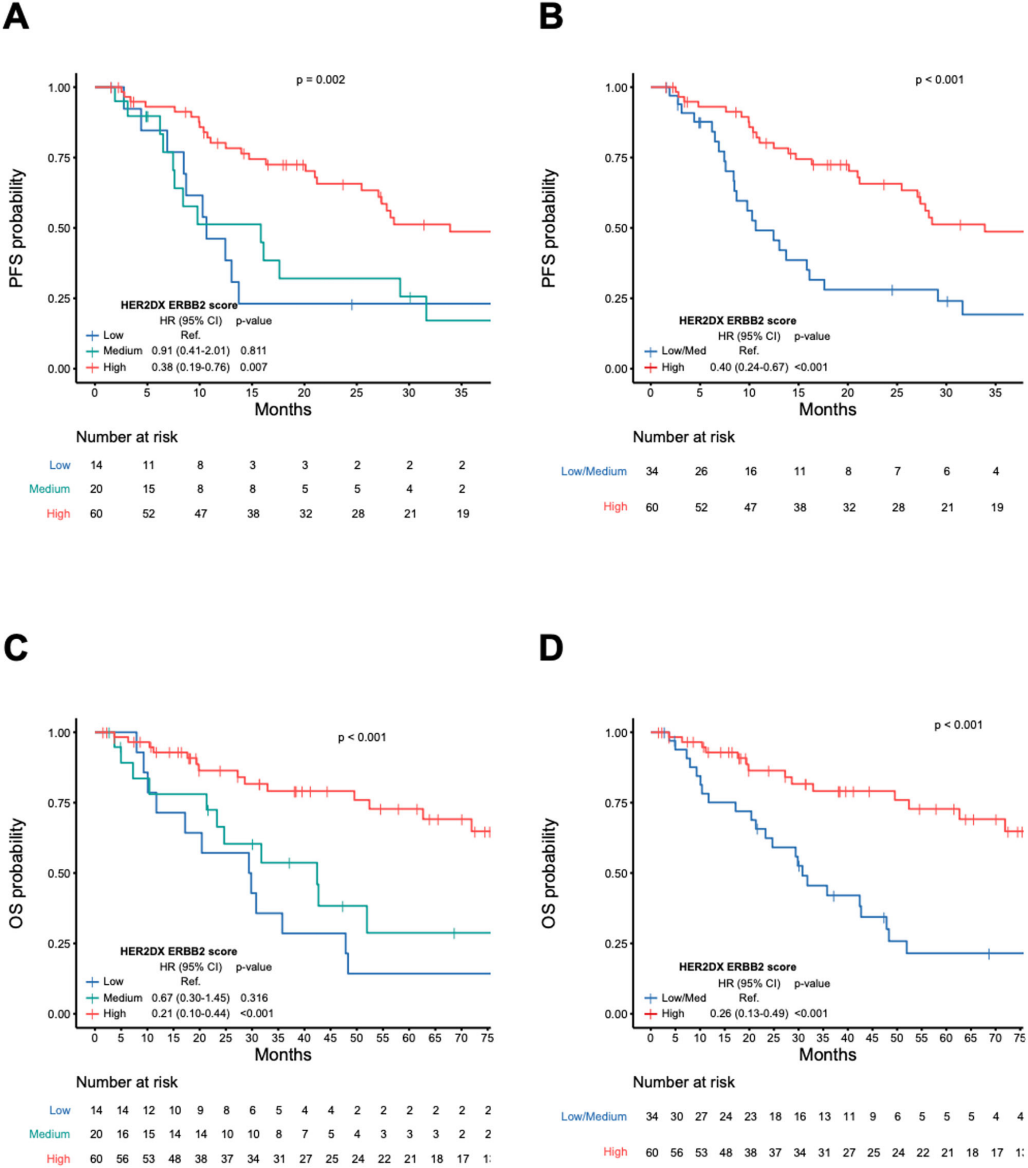
Association of HER2DX ERBB2 score and PFS and OS. **A** Association of HER2DX *ERBB2* mRNA score high, medium, and low groups and PFS. **B** Association of HER2DX *ERBB2* mRNA score high and low/medium groups and PFS. **C** Association of HER2DX *ERBB2* mRNA score high, medium, and low groups and OS. **D** Association of HER2DX *ERBB2* mRNA score high and low/medium groups and OS.

The median PFS and OS were 27.1 months (95% CI: 16.1–43.7 months) and 62.7 months (95% CI: 47.9–NR months), respectively. The HER2DX *ERBB2* mRNA score, when analyzed as a continuous variable, showed a significant association with both PFS (HR = 0.69 [95% CI: 0.53–0.88], *p* = 0.028) and OS (HR = 0.58 [95% CI: 0.43–0.77], *p* < 0.001) (Fig. 2A–D). When compared to the HER2DX *ERBB2*-low group, the HER2DX *ERBB2*-high group demonstrated significantly better outcomes, with a median PFS of 33.9 months versus 10.6 months (HR = 0.40 [95% CI: 0.24–0.69], *p* < 0.001) and a median OS that was not reached versus 30.8 months (HR = 0.26 [95% CI: 0.13–0.49], *p* < 0.001). Eight of nine (88.8%) patients without progression for over 5 years, and all patients without death over 8 years (*n* = 4) had HER2DX *ERBB2*-high scores.

In univariate analyses, clinical variables associated with PFS included de novo metastasis (HR = 0.56 [95% CI: 0.33–0.95], *p* = 0.031), visceral metastasis (HR = 2.1 [95% CI: 1.06–4.20], *p* = 0.033), bone-only disease (HR = 0.29 [95% CI: 0.11–0.81], *p* = 0.018), brain metastasis (HR = 2.4 [95% CI: 1.24–4.51], *p* = 0.009), and the number of metastatic sites (≥3 vs <3) (HR = 3.19 [95%CI: 1.86–5.46], p < 0.001) (Table 2), while the clinical variables associated with OS included visceral metastasis (HR = 2.5 [95% CI: 1.05–5.95], *p* = 0.031), brain metastasis (HR = 2.4 [95% CI: 1.2–5.05], *p* = 0.016), and the number of metastatic sites (≥3 vs <3) (HR = 2.8 [95% CI: 1.5–5.24], *p* = 0.001) (Table 3). Notably, HER2 IHC (3+ vs others) was significantly associated with better PFS (HR = 2.3 [95% CI: 1.32–3.96], *p* = 0.003) and OS (HR = 4.3 [95% CI: 2.25–8.06], *p* < 0.001).

**Table 2:** Uni- and multi-variable analyses for progression-free survival

Variable	Univariable			Multivariable		
	HR	95% CI	*p*-Value	HR	95% CI	*p*-Value
HER2DX ERBB2 score high vs low/med	0.40	0.24–0.67	<0.001	0.40	0.20–0.85	0.016
HER2 IHC 3+ vs others	0.44	0.25–0.76	0.003	1.22	0.57–2.62	0.615
Hormone receptor status (positive vs negative)	1.35	0.78–2.33	0.284	NA	NA	NA
De novo metastasis vs relapsed	0.56	0.33–0.95	0.031	0.70	0.39–1.18	0.17
Bone-only disease	0.29	0.11–0.81	0.018	0.52	0.14–1.93	0.329
Visceral metastasis	2.10	1.06–4.20	0.033	0.98	0.40–2.36	0.965
Brain metastasis	2.40	1.24–4.51	0.009	1.58	0.77–3.24	0.212
Metastatic sites ≥3 vs <3	3.19	1.86–5.46	<0.001	2.30	1.22–4.50	0.010

**Table 3:** Uni- and multi-variable analysis for overall survival

Variable	Univariable			Multivariable		
	HR	95% CI	*p*-Value	HR	95% CI	*p*-Value
HER2DX ERBB2 score high vs low/med	0.26	0.13–0.49	<0.001	0.40	0.17–0.85	0.018
HER2 IHC 3+ vs others	0.24	0.12–0.44	<0.001	0.47	0.21–1.07	0.072
Hormone receptor status (HR+ vs HR−)	1.28	0.67–2.45	0.46	NA	NA	NA
De novo metastasis vs relapsed	0.86	0.46–1.60	0.626	NA	NA	NA
Bone only disease	0.52	0.19–1.47	0.217	NA	NA	NA
Visceral metastasis	2.50	1.05–5.95	0.039	1.50	0.58–3.88	0.410
Brain metastasis	2.40	1.18–5.05	0.016	2.40	1.08–5.35	0.032
Metastatic sites ≥3 vs <3	2.80	1.50–5.24	0.001	1.60	0.74–3.29	0.244

In multivariable analyses, the HER2DX *ERRB2*-high group remained significantly associated with better PFS (HR = 0.41 [95% CI: 0.20–0.85], *p* = 0.016) and OS (HR = 0.38 [95% CI: 0.17–0.85], *p* = 0.018), while HER2 IHC (3+ vs others) lost its significance (PFS HR = 0.82 [95% CI: 0.38–1.76], *p* = 0.61) and (OS HR = 0.47 [95% CI: 0.21–1.07], p = 0.072)(Tables 2 and 3).

Finally, in this cohort, the ORR was 79.14%. The HER2DX *ERBB2* mRNA score was not associated with ORR (OR = 1.21 [95% CI: 0.89–1.65], *p* = 0.235). ORR by HER2DX *ERBB2* mRNA categories was 78.5% for the low group, 63.2% for the medium group, and 96.1% for the high group (OR HER2DX *ERBB2* high vs low/med = 2.37 [95% CI: 0.85–6.75], *p* = 0.100).

## Discussion

This study is, to our knowledge, the first to evaluate the association between the HER2DX *ERBB2* mRNA score and clinical outcomes in HER2+ advanced breast cancer patients treated with first-line THP chemotherapy. Our findings confirm that a higher HER2DX *ERBB2* mRNA score is significantly associated with improved PFS and OS, highlighting the prognostic value of HER2DX *ERBB2* mRNA expression in this setting.

Previous research by Brasó-Maristany et al.^21^ evaluated the role of HER2DX ERBB2 in metastatic breast cancer, demonstrating that higher *ERBB2* mRNA levels were significantly associated with better outcomes in patients treated with ado-trastuzumab emtansine (T-DM1). The study showed that higher *ERBB2* mRNA levels correlated with longer PFS and OS, regardless of HER2 IHC levels, hormone receptor status, brain metastasis, and line of therapy^21^. Our findings in the first-line THP setting further support the prognostic value of HER2DX *ERBB2* mRNA score, indicating that *ERBB2* mRNA is a robust biomarker across multiple lines of therapy in metastatic HER2+ breast cancer.

In our cohort, all patients who remained progression-free beyond 5 years and alive beyond 8 years had high HER2DX *ERBB2* mRNA scores, underscoring the clinical significance of this biomarker. Identifying long-term responders is crucial for optimizing treatment strategies. A high HER2DX *ERBB2* mRNA score could help clinicians personalize treatment by continuing THP in responders, while those with lower scores might benefit from more aggressive upfront treatments or alternative therapies. This approach could minimize unnecessary toxicity and improve the overall quality of life for patients.

The ongoing DESTINY-Breast09 trial could further reshape clinical practice in HER2+ metastatic breast cancer^6^. This phase III trial is recruiting 1134 patients and is comparing trastuzumab deruxtecan (T-DXd) to THP in the first-line setting, with PFS as the primary endpoint. Given T-DXd’s promising results in later lines of therapy^3–5^, it may become the new standard of care for first-line treatment if the trial proves its superiority. HER2DX *ERBB2* mRNA score could help clinicians stratify patients who are more likely to benefit from T-DXd, particularly those with lower *ERBB2* scores who may not respond well or do well to THP. The results of DESTINY-Breast09, combined with HER2DX *ERBB2* mRNA score stratification, could lead to more personalized treatment approaches and improved outcomes for HER2+ metastatic breast cancer patients. This is particularly relevant when considering differences in adverse events and treatment-related quality of life between T-DXd and THP. The HER2DX assay could play a crucial role in identifying long-term responders to THP, offering a potentially better-tolerated treatment option, particularly in the context of maintenance therapy. Furthermore, the recent results from the PATINA trial, which demonstrated the benefit of maintenance palbociclib following THP induction in HER2+/hormone receptor-positive disease, underscore the potential of THP as a viable treatment strategy, even as new evidence for T-DXd emerges^22^.

Interestingly, HER2 IHC was significantly associated with better OS in univariate analyses, but it lost significance when the HER2DX *ERBB2* mRNA score was included in multivariable analyses. This suggests that HER2DX *ERBB2* mRNA score may provide a more accurate assessment of HER2 status compared to IHC. While IHC has been the standard for HER2 assessment, it relies on subjective interpretation and can be affected by tissue fixation and antibody variability^20,23^. In contrast, HER2DX offers a standardized and quantitative measure of *ERBB2* mRNA^24^, potentially improving the precision of HER2 assessment.

The limitations of this study include its retrospective nature and relatively small sample size. Retrospective data collection introduces inherent biases, and the study’s conduct across two Spanish institutions may limit generalizability to other populations. Additionally, the lack of centralized HER2 testing could have introduced variability in IHC interpretation. Nonetheless, although not centralized, it was performed by expert breast pathologists. Although HER2 ISH testing was not consistently reported for all patients, we acknowledge its importance as the gold standard for HER2 status assessment. However, the lack of consistent FISH results reflects real-world clinical practice, where HER2 IHC 3+ is often sufficient for determining HER2 positivity. Future prospective studies with larger sample sizes and diverse populations are warranted to validate these findings. Another potential limitation is that tumor response was assessed by the treating physicians per clinical practice. With other tools, such as RECIST criteria, an association between HER2DX *ERBB2* mRNA score and ORR could be found. We acknowledge the lack of information regarding other biomarkers (e.g. *PIK3CA* mutatations) that have been associated with poorer outcomes in first-line HER2+ MBC^25^. Such biomarkers are not currently performed by clinical practice, precluding us from having access to this information. Finally, subsequent treatments after THP were not included in the analyses, potentially confounding the association with OS.

Despite these limitations, this study provides valuable insights into the utility of the HER2DX *ERBB2* mRNA score in HER2+ advanced breast cancer treated with THP. By offering a standardized, quantitative measure of HER2 status, HER2DX has the potential to improve upon conventional HER2 assessment methods and help tailor treatment strategies for patients.

In conclusion, the HER2DX *ERBB2* mRNA score is significantly associated with improved survival in patients receiving first-line THP for HER2+ advanced breast cancer. The ongoing DESTINY-Breast09 trial may further influence clinical practice, and incorporating HER2DX into treatment decision-making could optimize treatment strategies and enhance personalized care. Based on the results of this study, the HER2DX *ERBB2* mRNA score is also being tested in the CLEOPATRA phase III trial^1^, which led to the approval of pertuzumab.

## Methods

### Study population

We conducted a retrospective, multi-center observational study in patients treated with the standard taxane, trastuzumab, and pertuzumab (THP) regimen in the first-line setting for advanced HER2+ breast cancer between 2010 and 2024 from two Spanish institutions (Hospital Clinic of Barcelona, and Hospital Universitario 12 de Octubre in Madrid). Demographic and clinical–pathological characteristics were collected from electronic medical records. Eligible patients met the following criteria: histological confirmation of breast cancer with positive HER2 status, defined as either 3+ immunohistochemistry (IHC) or HER2 amplification by in situ hybridization (ISH); locally advanced disease not amenable to curative treatment or metastatic disease; and treatment with the THP regimen in the first-line setting. Both newly diagnosed and recurrent cases were included.

### Clinical HER2 status

HER2 status was locally assessed using IHC and/or ISH, following the ASCO/CAP guidelines at the time of diagnosis^20,23^. IHC was performed using an anti-HER-2/neu (4B5) Rabbit Monoclonal Primary Antibody kit (Ventana Medical Systems Inc., Oro Valley, AZ, USA). HER2 ISH was conducted using the FDA-approved XL ERBB2 (HER2/NEU) AMP (MetaSystems Probes, Altlußheim, Germany).

### RNA Extraction and HER2DX assay

Patients without sufficient archival tissue samples for HER2DX *ERBB2* mRNA score assay were excluded. One formalin-fixed paraffin-embedded (FFPE) tumor sample per patient was selected: if available, a biopsy of the metastatic site nearest in time to the start of THP was preferred. The standardized HER2DX assay was performed in a central lab (Barcelona, Spain). The HER2DX assay quantifies mRNA expression levels of 27 target genes and 5 normalization genes with constitutive expression (*GAPD, PUM1, ACTB, RPLP0*, and *PSMC4*). These 27 target genes are grouped into four distinct gene signatures: immune infiltration (*CD27, CD79A, HLA-C, IGJ, IGKC, IGL, IGLV3-25, IL2RG, CXCL8, LAX1, NTN3, PIM2, POU2AF1*, and *TNFRSF17*), tumor cell proliferation (*EXO1, ASPM, NEK2*, and *KIF23*), luminal differentiation (*BCL2, DNAJC12, AGR3, AFF3*, and *ESR1*), and HER2 amplicon expression (*ERBB2, GRB7, STARD3*, and *TCAP*). HER2DX integrates these molecular profiles with clinical characteristics, including tumor and nodal stage, to generate three key scores: long-term prognosis (risk score), likelihood of achieving a pathological complete response (pCR score), and *ERBB2* mRNA expression (*ERBB2* score)^24^. The HER2DX *ERBB2* score was calculated based on the *ERBB2* mRNA levels. Pre-established cutoffs, derived from prior validation studies, were used to categorize scores as low, medium, or high^15^.

### Endpoints and statistical analyses

The primary objective was to assess the association between HER2DX *ERBB2* mRNA score, as both a continuous variable and categorized (low, medium, high), with PFS and OS. Secondary objectives included evaluating the association of HER2DX *ERBB2* mRNA score with the overall response rate (ORR) according to the physician´s local report. PFS was defined as the time from the initiation of THP treatment to disease progression or death, whichever occurred first. OS was defined as the time from the start of THP treatment to death from any cause. Missing data were not imputed, and patients with incomplete clinical data were excluded from the respective analyses. Univariate and multivariable Cox proportional hazard regression analyses were performed to assess associations with PFS and OS, while logistic regression was used to evaluate ORR. Sensitivity analyses were performed to ensure the robustness of the findings, and proportional hazard assumptions were tested for the Cox models. Statistical analyses were conducted using R v4.2.2, with a two-sided *p*-value < 0.05 considered statistically significant.

## Supplementary information

Supplementary Figure 1

## Data Availability

Data are available upon reasonable request.
