# Supplementary material for: HER2DX ERBB2 mRNA score in first-line advanced HER2-positive breast cancer treated with chemotherapy, trastuzumab, and pertuzumab
Source: NPJ Breast Cancer. 2025 Apr 25;11:37. doi: 10.1038/s41523-025-00753-8 (PMC12032064; doi:10.1038/s41523-025-00753-8)
Supplement: Supplementary file 1 — Supplementary Figure 1 [file 41523_2025_753_MOESM1_ESM.docx]

**
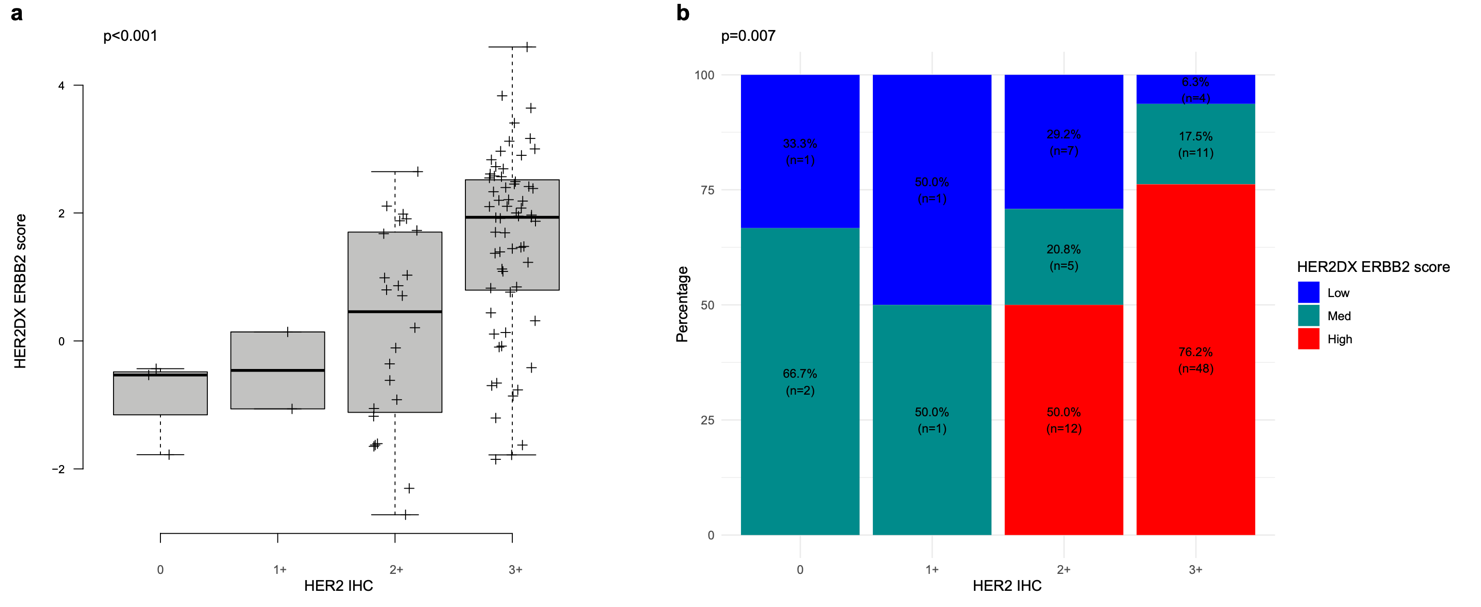
**

**Supplementary Figure 1.** Association of HER2DX ERBB2 score and HER2 IHC. **(A)** Correlation of HER2DX ERBB2 mRNA score as continuous variable and HER2 IHC categories (0, 1+, 2+, 3+). **(B)** Distribution of HER2DX ERBB2 mRNA score high, medium and low groups and HER2 IHC categories (0, 1+, 2+, 3+).
